# The association between first and second wave COVID-19 mortality in Italy

**DOI:** 10.1186/s12889-021-12126-4

**Published:** 2021-11-11

**Authors:** Marco Vinceti, Tommaso Filippini, Kenneth J. Rothman, Silvia Di Federico, Nicola Orsini

**Affiliations:** 1 Environmental, Genetic and Nutritional Epidemiology Research Center (CREAGEN), Section of Public Health, Department of Biomedical, Metabolic and Neural Sciences, University of Modena and Reggio Emilia, Modena, Italy; 2 Department of Epidemiology, Boston University School of Public Health, Boston, MA US; 3 RTI Health Solutions, Research Triangle Park, Raleigh, NC US; 4 Department of Global Public Health, Karolinska Institutet, Stockholm, Sweden

**Keywords:** COVID-19, Epidemiology, Mortality, Public health, SARS-CoV-2, Waves

## Abstract

**Background:**

The relation between the magnitude of successive waves of the COVID-19 outbreak within the same communities could be useful in predicting the scope of new outbreaks.

**Methods:**

We investigated the extent to which COVID-19 mortality in Italy during the second wave was related to first wave mortality within the same provinces. We compared data on province-specific COVID-19 2020 mortality in two time periods, corresponding to the first wave (February 24–June 30, 2020) and to the second wave (September 1–December 31, 2020), using cubic spline regression.

**Results:**

For provinces with the lowest crude mortality rate in the first wave (February–June), i.e. < 22 cases/100,000/month, mortality in the second wave (September–December) was positively associated with mortality during the first wave. In provinces with mortality greater than 22/100,000/month during the first wave, higher mortality in the first wave was associated with a lower second wave mortality. Results were similar when the analysis was censored at October 2020, before the implementation of region-specific measures against the outbreak. Neither vaccination nor variant spread had any role during the study period.

**Conclusions:**

These findings indicate that provinces with the most severe initial COVID-19 outbreaks, as assessed through mortality data, faced milder second waves.

## Background

The COVID-19 pandemic in many countries has been characterized by waves of infection. These recurrent local exacerbations of the pandemic present an opportunity to study the spread of the virus within a population. Key features of the pandemic are still not well understood, such as the susceptibility of the population to subsequent waves after the first outbreak, the threshold for herd immunity, the role of superspreaders [1–7] as well as of meteorological and environmental factors [8–11].

We compared the two COVID-19 waves within Italy, where the geographical distribution of the SARS-CoV-2 infection spread and the COVID-19 incidence was uneven during the first wave of the pandemic [12]. From an analysis within provinces of the official case counts during the first and second wave of SARS-CoV-2 infection, we found evidence of an association in the occurrence between the two periods. When incidence in the first wave was low (< 500 cases/100,000/day), the second wave incidence tended to be positively correlated, whereas a high first-wave incidence was strongly and inversely correlated with the second wave incidence. This observation raised the possibility that greater spread at the beginning of the pandemic could have induced some protection at the population level, resulting in a milder second wave, despite low levels (10–20%) of anti-SARS-CoV-2 antibody seroprevalence even in the areas most severely hit.

One problem with our earlier finding is that the incidence of SARS-CoV-2 infection is difficult to measure. It depends on the severity of the clinical course, the availability of molecular tests and the policy underlying their use at the population level. In Italy, for instance, the implementation of nasopharyngeal swabs and molecular tests to detect SARS-CoV-2 infection in the first wave was restricted primarily to symptomatic patients with suspected COVID-19. In the summer of 2020, however, the public-health policy was modified to extend testing to contacts of cases and to individuals without clinical symptoms, nearly tripling the number of daily molecular tests from the first to the second wave time periods [13]. An additional change of policy, starting on November 6, 2020, during the second wave, was the mandatory adoption of differential area-specific public-health measures [14, 15].

The area-specific relation between the two waves may be better assessed by a more objective measures of COVID-19 spread such as mortality rate, instead of incidence rates that are subject to swab testing policy and availability. Recently, COVID-19 death data for Italy in 2020 have become available with greater geographic detail, with figures available for the 107 provinces rather than the 20 larger regions within Italy. We used these mortality data to revisit the relation of the dynamics of the first and second waves of the outbreak.

## Methods

### Study population and outcome

We used data made freely available from public sources. We accessed 2020 monthly province-specific COVID-19 deaths as reported by the Italian National Institute of Statistics – ISTAT [16]. Provinces are the administrative entities that are intermediate between smaller municipalities and larger regions within Italy. Within 2020, we examined two time frames: we considered the first wave to be the period from the pandemic onset (February 24) to June 30, when the number of new infection cases had substantially dropped after the March–April peak. From July to the end of August the number of new cases remained low, mostly related to screening of subjects returning from vacation with swab testing. Cases increased again beginning in early September. Therefore, we considered the second wave to be the period from September 1 to December 31, 2020. During these periods, neither circulation of virus variants nor implementation of SARS-CoV-2 vaccination at the population level had yet started in Italy. Only a handful of Italians had been vaccinated by the end of December, but large scale administration started in January 2021 [17, 18].

Based on these data and the Italian population at January 1, 2020, and January 1, 2021 available at the ISTAT website [19], we computed wave-specific COVID-19 mortality during the first and second waves. For the former period, we used as reference population data the provincial population size as of January 1, 2020. For the second period and for the overall 2020 mortality we averaged the population size at January 1, 2020 and January 1, 2021 for each province. We retrieved the seroprevalence data for each Italian region and province determined through a national survey made available by ISTAT and the National Ministry of Health with reference to the period May 25, 2020 through July 15, 2020 [20].

### Data analysis

We examined the relation between first and second wave province-specific crude mortality rates from COVID-19 by modeling the mean mortality rate using a restricted cubic spline regression [21], a model that fits a curvilinear pattern to the data as previously reported [22–24]. In particular, we fitted a restricted cubic spline model that weighted provincial data by population size, performing both a crude analysis and adjusting for potential confounders. Variables included in the model were aging index (i.e. a ratio between resident population aged ≥65 years and those aged ≤14 years), percentage commuting outside the municipality of residence on a daily basis, and percentage of dwellings occupied by only one resident (available at a provincial level from the National Institute of Statistics [19]). In the regression model, we used three knots at fixed percentiles (10th, 50th and 90th) of the first wave distribution, and computed a pointwise 95% confidence interval (CI) [21, 25]. We used ‘mkspline’, ‘regress’, and ‘xbrcsplinei’ routines of Stata software (v17.0, College Station-TX, 2021) for all analyses.

## Results

**Fig. 1:**
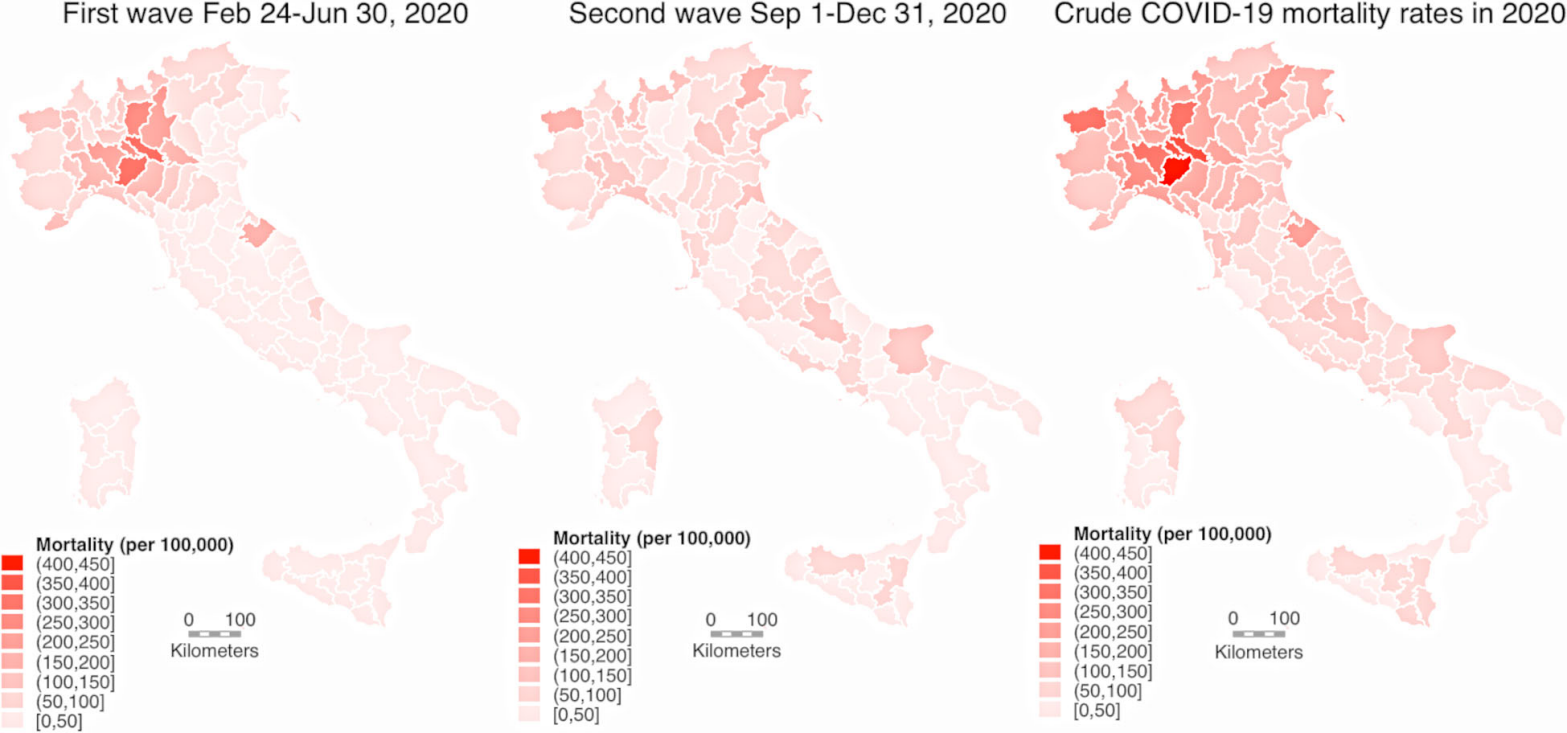
COVID-19 mortality in the Italian provinces in the first two pandemic waves and total 2020 mortality

Table 1 and Fig. 1 lists province-specific mortality rates in the investigated periods. National cumulative mortality (cumulative incidence of death) during the first wave averaged 58.2/100,000 persons, ranging from 6.5/100,000 persons in the Southern Italy, Basilicata region, to 165/100,000 persons in Northern Italy, Lombardy region. Corresponding figures for the second wave period were 67.6/100,000 persons for the national average, ranging from 19.4/100,000 in the Calabria region to 192/100,000 persons in the Aosta Valley region. In particular, seven provinces, five of the in Lombardy region (Bergamo, Brescia, Cremona, Lodi, and Pavia) and two in Emilia-Romagna region (Parma and Piacenza) experienced the highest mortality rates during the first wave. Second wave mortality was lower than first wave, always ≤100/100,000 persons and generally lower than the regional or national averages. The provinces with the highest first wave mortality, namely Piacenza (with the overall highest mortality in Italy with 333/100,000 persons), Cremona, Lodi and Bergamo, experienced the highest absolute decrease in mortality rates.

**Table 1:** Number of SARS-CoV-2 cases, COVID-19 deaths and COVID-19 mortality rates (deaths/100,000/wave timeframe) in the 1st and 2nd waves in 2020 divided by province

Province/ Region	Population Jan 1, 2020	Population Jan 1, 2021	Cases 1st wave	Cases 2nd wave	Seroprev. (%)	Deaths 1st wave	Deaths 2nd wave	Mortality 1st wave	Mortality 2nd wave	All Deaths	Overall Mortality
**Aosta Valley**	**125,501**	**123,895**	**1195**	**5771**	**3.72**	**145**	**239**	**116**	**192**	**384**	**308**
Aosta	125,501	123,895	1195	5771	3.72	145	239	116	192	384	308
**Lombardy**	**10,103,969**	**9,966,992**	**91,813**	**368,273**	**7.35**	**16,633**	**8321**	**165**	**82.9**	**25,120**	**250**
Bergamo	1,116,384	1,099,621	14,375	12,873	24.3	3137	193	281	17.4	3347	302
Brescia	1,268,455	1,247,583	15,626	25,468	7.63	2686	422	212	33.5	3117	248
Como	603,828	594,671	4093	29,531	2.00	587	794	97.2	133	1388	232
Cremona	358,347	351,698	6612	7664	19.7	1130	123	315	34.6	1261	355
Lecco	337,087	332,593	2831	10,303	6.66	481	236	143	70.5	724	216
Lodi	230,607	225,885	3570	6936	7.10	679	140	294	61.3	826	362
Mantua	411,062	403,585	3496	12,260	6.57	684	288	166	70.7	975	239
Milan	3,279,944	3,249,821	24,379	147,720	3.95	4252	3197	130	97.9	7509	230
Monza/Brianza	878,267	867,421	5772	42,090	4.52	979	895	112	103	1884	216
Pavia	546,515	534,951	5568	18,869	5.95	1241	543	227	100	1806	334
Sondrio	180,941	179,234	1584	6954	5.30	212	201	117	112	415	230
Varese	892,532	879,929	3907	47,605	1.71	565	1289	63.3	145	1868	211
**Veneto**	**4,907,704**	**4,852,453**	**18,937**	**227,276**	**1.92**	**2028**	**4960**	**41.3**	**102**	**7079**	**145**
Belluno	201,972	199,599	1191	13,369	1.88	114	330	56.4	164	445	222
Padua	939,672	929,520	3954	41,651	2.32	318	608	33.8	65.1	946	101
Rovigo	233,386	229,652	444	6932	2.39	36	204	15.4	88.1	240	104
Treviso	888,309	878,070	2673	45,715	1.89	322	783	36.2	88.7	1112	126
Venice	851,663	842,942	2682	35,612	1.68	299	863	35.1	102	1185	140
Verona	930,339	922,291	5127	44,073	2.23	586	1195	63.0	129	1789	193
Vicenza	862,363	850,379	2866	39,924	1.33	353	977	40.9	114	1362	159
**Emilia-Romagna**	**4,467,118**	**4,445,549**	**28,061**	**137,052**	**2.90**	**4353**	**3431**	**97.4**	**77.0**	**7825**	**176**
Bologna	1,017,806	1,019,539	5229	32,314	2.33	732	936	71.9	91.9	1681	165
Ferrara	344,840	341,967	1044	7886	0.72	173	222	50.2	64.6	396	115
Forlì-Cesena	394,833	393,556	1740	10,213	1.04	196	164	49.6	41.6	362	91.8
Modena	707,292	704,672	3873	25,945	1.10	480	601	67.9	85.1	1085	154
Parma	453,930	453,604	3657	8701	5.84	901	215	199	47.4	1119	247
Piacenza	287,236	284,075	4428	10,187	9.54	956	252	333	88.2	1213	425
Ravenna	389,634	386,309	1030	11,337	1.18	81	430	20.8	111	514	133
Reggio nell’Emilia	531,751	526,349	4913	18,248	4.45	581	306	109	57.8	891	168
Rimini	339,796	335,478	2147	12,221	2.79	253	305	74.5	90.3	564	167
**Piedmont**	**4,341,375**	**4,273,210**	**30,989**	**162,730**	**3.45**	**4029**	**3537**	**92.8**	**82.1**	**7583**	**176**
Alessandria	419,037	411,922	4063	13,240	2.08	659	470	157	113	1134	273
Asti	213,216	209,648	1874	7960	2.13	249	217	117	103	466	220
Biella	174,384	171,838	1046	5748	6.59	194	112	111	64.7	306	177
Cuneo	586,568	582,353	2862	24,081	0.87	373	494	63.6	84.5	869	149
Novara	368,040	362,199	2792	12,443	5.21	367	272	99.7	74.5	642	176
Turin	2,252,379	2,212,996	15,889	87,788	3.58	1844	1712	81.9	76.7	3561	160
Verbano-Cusio-Ossola	157,455	155,065	1140	5515	9.05	132	122	83.8	78.1	254	163
Vercelli	170,296	167,189	1323	5955	3.52	211	138	124	81.8	351	208
**Trentino-South Tyrol**	**1,074,819**	**1,078,460**	**7502**	**43,303**	**9.93**	**693**	**1041**	**64.5**	**96.7**	**1734**	**161**
Bolzano	532,080	533,715	2639	26,559	2.95	288	504	54.1	94.6	792	149
Trento	542,739	544,745	4863	16,744	3.42	405	537	74.6	98.8	942	173
**Friuli-Venezia Giulia**	**1,211,357**	**1,198,753**	**3308**	**45,651**	**1.02**	**362**	**1426**	**29.9**	**118**	**1794**	**149**
Gorizia	139,206	136,809	216	5904	0.12	5	104	3.6	75.4	109	79.0
Pordenone	312,619	309,058	702	9792	1.88	68	291	21.8	93.6	360	116
Trieste	233,276	229,470	1393	9107	0.59	209	270	89.6	117	483	209
Udine	526,256	523,416	997	20,848	0.93	80	761	15.2	145	842	160
**Liguria**	**1,543,127**	**1,509,805**	**9473**	**46,958**	**3.24**	**1563**	**1276**	**101**	**86.3**	**2851**	**187**
Genoa	835,829	816,916	5573	29,304	3.61	943	853	113	103	1802	218
Imperia	213,919	208,585	1494	4806	2.39	231	79	108	37.4	312	148
La Spezia	219,196	215,538	860	7142	1.89	159	189	72.5	86.9	349	161
Savona	274,183	268,766	1546	5706	3.83	230	155	83.9	57.1	388	143
**Tuscany**	**3,722,729**	**3,668,333**	**9779**	**108,429**	**0.90**	**1088**	**2491**	**29.2**	**67.4**	**3604**	**97.5**
Arezzo	341,766	336,870	676	9779	1.23	47	168	13.8	49.5	218	64.2
Florence	1,004,298	986,001	3192	29,864	0.53	401	839	39.9	84.3	1249	126
Grosseto	220,785	218,538	396	3708	1.18	28	73	12.7	33.2	102	46.4
Livorno	333,509	329,590	477	8079	0.56	62	195	18.6	58.8	260	78.4
Lucca	388,678	380,676	1351	11,010	0.42	151	192	38.8	49.9	347	90.2
Massa and Carrara	193,934	189,841	1051	6442	0	153	179	78.9	93.3	336	175
Pisa	422,310	416,425	930	15,667	1.55	91	341	21.5	81.3	433	103
Pistoia	293,059	290,819	747	9640	0.96	76	199	25.9	68.2	275	94.2
Prato	258,152	256,047	532	9790	1.02	47	203	18.2	79.0	250	97.2
Siena	266,238	263,526	427	4450	2.17	32	102	12.0	38.5	134	50.6
**Umbria**	**880,285**	**865,013**	**1385**	**26,064**	**0.670**	**80**	**530**	**9.10**	**60.7**	**610**	**69.9**
Perugia	655,403	643,311	1008	19,843	0.71	51	369	7.80	56.8	420	64.7
Terni	224,882	221,702	377	6221	0.55	29	161	12.9	72.1	190	85.1
**Marches**	**1,518,400**	**1,501,406**	**6549**	**33,194**	**2.59**	**987**	**720**	**65.0**	**47.7**	**1709**	**113**
Ancona	469,750	465,023	1875	9711	2.16	218	185	46.4	39.6	403	86.2
Ascoli Piceno	206,363	204,575	290	4790	4.95	12	125	5.8	60.8	137	66.7
Fermo	173,004	170,248	473	4337	2.16	67	69	38.7	40.2	137	79.8
Macerata	312,146	307,421	1154	7851	2.16	145	159	46.5	51.3	305	98.5
Pesaro and Urbino	357,137	354,139	2757	6505	4.95	545	182	153	51.2	727	204
**Lazio**	**5,865,544**	**5,720,796**	**8010**	**148,533**	**1.00**	**863**	**2815**	**14.7**	**48.6**	**3717**	**64.2**
Frosinone	485,241	473,467	663	12,990	0.19	79	162	16.3	33.8	241	50.3
Latina	576,655	561,139	607	13,625	0.50	44	294	7.6	51.7	340	59.8
Rieti	154,232	151,668	411	4565	3.00	41	149	26.6	97.4	191	125
Rome	4,333,274	4,227,588	5872	108,988	1.05	672	2016	15.5	47.1	2724	63.6
Viterbo	316,142	306,934	457	8365	1.52	27	194	8.5	62.3	221	70.9
**Abruzzo**	**1,305,770**	**1,285,256**	**3261**	**31,124**	**1.29**	**461**	**794**	**35.3**	**61.3**	**1264**	**97.6**
Chieti	383,189	376,397	818	6284	1.40	131	136	34.2	35.8	270	71.1
L’Aquila	296,491	292,356	225	10,604	0.54	11	350	3.7	119	362	123
Pescara	318,678	314,689	1586	5447	1.69	239	116	75.0	36.6	360	114
Teramo	307,412	301,814	632	8789	1.48	80	192	26.0	63.0	272	89.3
**Molise**	**302,265**	**296,547**	**426**	**5971**	**0.81**	**28**	**175**	**9.3**	**58.4**	**203**	**67.8**
Campobasso	218,679	214,629	364	3829	0.66	22	110	10.1	50.8	132	60.9
Isernia	83,586	81,918	62	2142	1.19	6	65	7.2	78.5	71	85.8
**Campania**	**5,785,861**	**5,679,759**	**4648**	**182,462**	**0.89**	**517**	**2915**	**8.9**	**50.8**	**3447**	**60.1**
Avellino	413,926	405,963	552	8289	0	62	143	15.0	34.9	206	50.3
Benevento	274,080	269,233	209	4423	0	19	137	6.9	50.4	156	57.4
Caserta	922,171	911,606	543	33,741	1.48	53	540	5.7	58.9	596	65.0
Naples	3,082,905	3,017,658	2652	111,294	1.04	314	1811	10.2	59.4	2133	69.9
Salerno	1,092,779	1,075,299	692	24,715	0.31	69	284	6.3	26.2	356	32.8
**Apulia**	**4,008,296**	**3,926,931**	**4502**	**84,951**	**0.88**	**566**	**2037**	**14.1**	**51.3**	**2614**	**65.9**
Bari	1,249,246	1,222,818	1491	33,237	1.50	153	636	12.2	51.5	793	64.2
Barletta-Andria-Trani	388,390	382,685	380	10,058	0.77	66	295	17.0	76.5	361	93.6
Brindisi	390,456	382,454	659	5795	0.85	67	100	17.2	25.9	167	43.2
Foggia	616,310	601,419	1170	18,639	1.02	161	655	26.1	108	819	135
Lecce	791,122	777,507	521	6420	0.01	85	114	10.7	14.5	200	25.5
Taranto	572,772	560,048	281	10,802	0.67	34	237	5.9	41.8	274	48.4
**Basilicata**	**556,934**	**547,579**	**400**	**10,055**	**0.72**	**36**	**214**	**6.5**	**38.8**	**251**	**45.4**
Potenza	360,936	354,122	189	6739	0.83	27	156	7.5	43.6	184	51.5
Matera	195,998	193,457	211	3316	0.50	9	58	4.6	29.8	67	34.4
**Calabria**	**1,924,701**	**1,877,728**	**1179**	**22,191**	**0.51**	**129**	**368**	**6.7**	**19.4**	**497**	**26.1**
Catanzaro	354,851	346,514	214	3134	0.40	31	49	8.7	14.0	80	22.8
Cosenza	700,385	684,786	468	6676	0.78	48	176	6.9	25.4	224	32.3
Crotone	170,718	166,617	119	2065	0.11	10	35	5.9	20.8	45	26.7
Reggio di Calabria	541,278	526,586	294	8586	0.18	29	81	5.4	15.2	110	20.6
Vibo Valentia	157,469	153,225	84	1730	1.12	11	27	7.0	17.4	38	24.5
**Sicily**	**4,968,410**	**4,840,876**	**3056**	**89,352**	**0.37**	**342**	**2390**	**6.9**	**48.7**	**2747**	**56.0**
Agrigento	429,611	419,847	135	3651	0.19	24	107	5.6	25.2	131	30.8
Caltanissetta	260,779	252,803	186	3733	0	18	81	6.9	31.5	99	38.6
Catania	1,104,974	1,066,765	779	26,464	0.26	103	849	9.3	78.2	957	88.1
Enna	162,368	158,183	438	2866	0	34	76	20.9	47.4	110	68.6
Messina	620,721	609,223	474	10,246	0.32	59	136	9.5	22.1	195	31.7
Palermo	1,243,328	1,214,291	500	24,929	0.89	43	665	3.5	54.1	708	57.6
Ragusa	321,215	314,950	87	6490	0.30	6	161	1.9	50.6	168	52.8
Siracusa	397,037	386,451	321	5112	0.14	47	162	11.8	41.4	218	55.6
Trapani	428,377	418,363	136	5861	0	8	153	1.9	36.1	161	38.0
**Sardinia**	**1,630,474**	**1,598,225**	**1366**	**28,920**	**0.50**	**145**	**712**	**8.9**	**44.1**	**858**	**53.1**
Cagliari	430,914	420,117	253	6573	0.38	19	161	4.4	37.8	180	42.3
Nuoro	206,843	202,951	78	6055	0.24	12	123	5.8	60.0	135	65.9
Oristano	156,078	153,226	61	2416	0.43	8	51	5.1	33.0	59	38.2
Sassari	489,634	481,052	875	8997	0.78	90	247	18.4	50.9	338	69.6
South Sardinia	347,005	340,879	99	4879	0.42	16	130	4.6	37.8	146	42.4
**Italy**	**60,244,639**	**59,257,566**	**235,839**	**1,808,260**	**2.49**	**35,048**	**40,392**	**58.2**	**67.6**	**75,891**	**127**

**Fig. 2:**
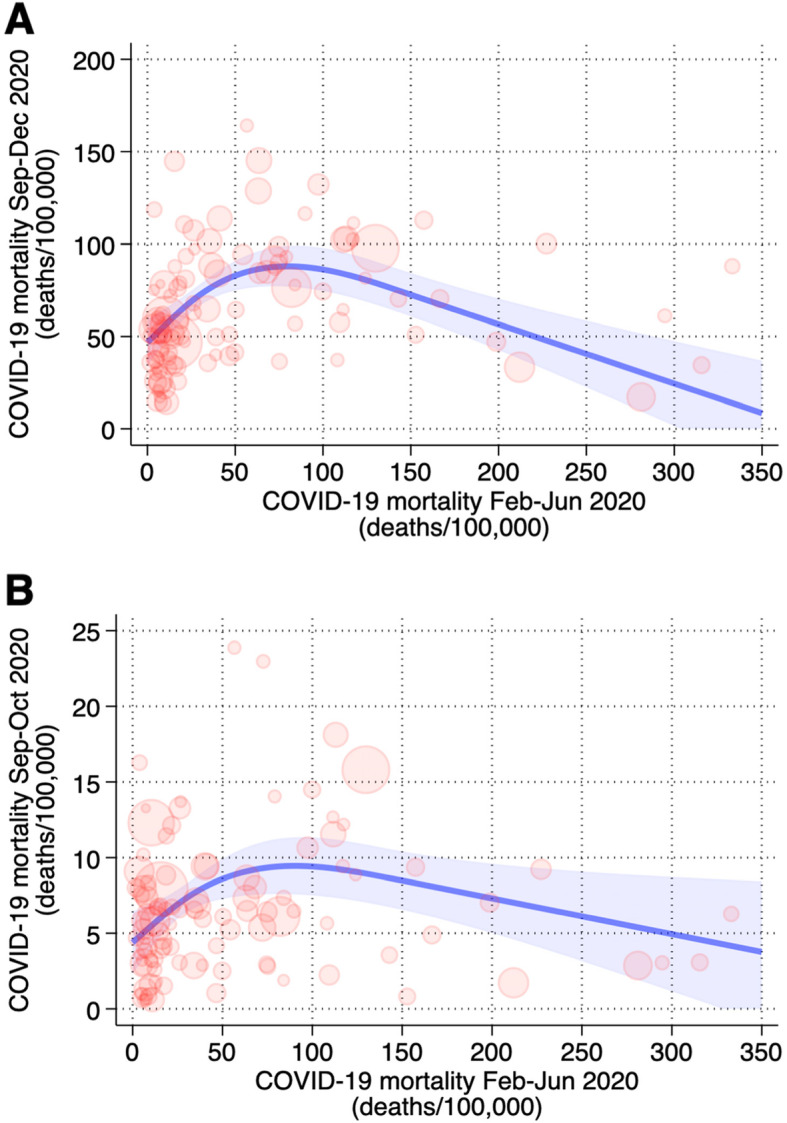
Population-weighted cubic spline regression analysis of the relation between first (from February 24 to June 30, 2020) and (A) second wave (from September 1 to December 31, 2020) mortality rates of SARS-CoV-2 infection in Italian provinces or (B) second wave before the beginning of mobility restrictions (from September 1 to October 31, 2020) mortality rates of SARS-CoV-2 infection in Italian provinces adjusted for aging index, percentage commuting outside the municipality of residence on a daily basis, and percentage of dwellings occupied by only one resident. Shadow area is the 95% confidence interval of the predicted mean mortality rate (solid line); circles are province-specific values weighted for population size

In the spline analysis, the relation between mortality in the first and second wave was U-shaped (Fig. 2), with a direct association between the estimates of the two waves up to 88 deaths /100,000, and above that an inverse pattern, with low second wave figures for those provinces with the highest rates during the first wave (88 through 192 deaths/100,000). The latter corresponded to a mortality rate of 22/100,000/month, as only 5 deaths were recorded in February 2020 following the first disease diagnosis in Italian residents on February 20. Crude analysis, ignoring control of potential confounders such as proportion elderly, living alone, and degree of mobility, gave similar results. When we repeated the main analysis by limiting the second wave to September–October 2020, i.e. by removing the November–December period when region-specific public health and social distancing policies were first allowed and implemented, results were not substantially changed, though mortality in this truncated period was much lower (Fig. 2).

## Discussion

Italy was the first country where the SARS-CoV-2 infection swept out of control, following the initial outbreak in China, where it was kept under control and geographically confined. The early arrival of the pandemic in Italy allowed a longer time frame to monitor and study the behavior of the epidemic. Italy did not allow the adoption of area-specific policies to curtail the outbreak until November, using instead a nationwide approach imposed by the Italian Government. It has been suggested recently that severity of COVID-19 pneumonia in hospitalized subjects was lower during the second wave [26], possibly due to SARS-CoV-2 genomic variation [27]. However, during the time frame of this study there was no evidence of spread of SARS-CoV-2 variants [17], thus avoiding any confounding effect from differential geographic spread of viral lineages with different transmission or virulence features. In addition, recent assessment of fatality rates between first and second waves in Italy showed that they were comparable [28]. Nonetheless, we cannot rule out that improvement in COVID-19 treatment may have decreased mortality during the pandemic, especially in the second wave, despite the lack of fully effective treatments [29–31]. Finally, vaccination had no role in curbing either of the two waves analyzed here, since almost no vaccine doses were administered in Italy in 2020. Indeed, the vaccination campaign had not begun yet in the investigated period, apart from a handful of ‘demonstration’ vaccinations administered to health professionals on December 27, 2020 at the national hub of the Spallanzani Hospital in Rome. Large scale administration started in January 2021 [32].

In contrast to our earlier analysis that relied on SARS-CoV-2 infection incidence, when area-specific mortality was still unavailable, we used mortality from COVID-19 to gauge the severity of the waves.

Mortality is a far more reliable indicator than incidence for documenting the actual trends of the pandemic, because the detection and notification of newly-infected cases from the general population depends on the availability and implementation of molecular testing, which in turn depends on health authority guidelines and policies [33]. Therefore, the number and type of individuals to be subject to SARS-CoV-2 testing may differ considerably across geographic areas or time periods independently of real differences in incidence, due to changing referral guidelines (such as the presence of COVID-19 symptoms) and availability of molecular tests. In Italy, first wave health policy allowed COVID-19 molecular testing only for COVID-19 symptomatic individuals, while in the second wave much more testing occurred, with systematic extension to asymptomatic or weakly symptomatic individuals and therefore a great increase in number of notifications. As a consequence, the ‘official’ SARS-CoV-2 infection incidence strongly increased in the second wave. This increase, however, could have been an artifact due to the aforementioned changes in testing policy and availability, especially in light of the roughly comparable numbers of deaths in the two waves. However, incidence is less reliable compared to mortality in documenting the real trend of the pandemic, also due to its inherent differences compared with SARS-CoV-2 infection incidence and with the hybrid pattern of the related assessments at the population level, due to changes in molecular testing availability and policy.

We were able to confirm the previously reported incidence patterns for SARS-CoV-2 in Italy during 2020 [12]. The key findings were a positive association between the two waves when the epidemic was not particularly strong in the first part of the year, and a negative association when the first wave was severe. This finding was similar when using a different time frame for the second wave to account for the possible influence of local policies. Local policies were permitted beginning as of November 6, 2020. In addition, the pattern we investigated is based on ‘real’ data, compared to studies presenting predictive modeling [34, 35], thus strengthening our findings.

Provinces that were relatively lightly affected by the first wave had a similar experience in the second wave, with rates closely mirroring those experienced during the first phase of the outbreak. In contrast, the Italian provinces severely hit by the first wave, such as Bergamo, Lodi, Cremona and Piacenza, experienced a marked reduction of mortality in the second wave, and presumably in the spread of COVID-19. The reasons for such an inverse pattern, however, were not directly identifiable through this study and may only be hypothesized. One possibility is a higher likelihood of immunity for superspreaders and individuals with high mobility and propensity to transmission during the first wave [36]. Another possible explanation is the development of an additional cellular immunity against SARS-CoV-2 in the most severely hit population during the first wave [37, 38]. The provinces most severely affected by the first wave showed a population-based seroprevalence in the 10–25% range, far below the estimated threshold for herd immunity of 50–70% [39]. It is possible that the prevalence of immunity, despite being below the herd immunity threshold, was high enough in provinces with a severe first wave to interact with behavioral factors and lessen the intensity of the second wave.

## Data Availability

We used data made freely available from public sources. All data generated or analysed during this study are included in this published article.
